# The Practice in the Free Churches

**Published:** 1917-01-20

**Authors:** 


### The Practice in the Free Churches.

A PREACHER who is visiting a large number of Baptist Churches writes: "The practice in the Free Churches is to make a collection at every Service, generally for the maintenance of public worship. As you know there is no tithe or endowment in most cases. In the majority of cases every Sunday offering is needed for this purpose. In the minority of cases it is customary to give up a certain number of Sundays to the objects of the denomination for which the Church stands. In the case of the Baptists these would be the Missionary Society, the

Sustentation Fund, etc. In almost every case where appeals are made for objects with which the Church is in sympathy, but which are not purely denominational, it is customary in making the appeal to say : ' Everything that is over and above your usual gifts will be given to the hospitals ' or to the Red Cross, or whatever the case may be. The alternative is to have the ordinary collection for the Church's own needs, which is generally taken in the middle of the Service, and then a retiring collection for special objects."

